# Epidemiology of neuroendocrine neoplasmas in Japan: based on analysis of hospital-based cancer registry data, 2009 – 2015

**DOI:** 10.1186/s12902-022-01016-4

**Published:** 2022-04-20

**Authors:** Tomonobu Koizumi, Kengo Otsuki, Yuriko Tanaka, Shintaro Kanda

**Affiliations:** Department of Hematology and Medical Oncology, Shinshu University School of Medicine, 3-1-1 Asahi, Matsumoto, Nagano, 390-8621 Japan

**Keywords:** Epidemiology, Carcinoid, Rare tumor, Pancreatic neuroendocrine tumor, Cancer registry

## Abstract

**Purpose:**

Neuroendocrine neoplasms are rare disease and could originate from throughout the body, however, there have been little epidemiological studies in Japan, especially the organ distribution. This study was to examine the epidemiological information of neuroendocrine neoplasms in the Japanese population using data from a hospital-based cancer registry.

**Methods:**

Using data from the national database of hospital-based cancer registries, we examined the organ distribution, the stage and initial treatment of neuroendocrine neoplasms newly diagnosed and treated in designated and non-designated cancer care hospitals between 2009 and 2015. In the present study, neuroendocrine neoplasms consisted of neuroendocrine tumors and carcinoma.

**Results:**

A total of 33,215 (17,485 neuroendocrine carcinomas and 15,730 neuroendocrine tumors) cases were diagnosed. The majority in neuroendocrine carcinoma occur in lung (31.1%) followed in decreasing frequency by stomach (12.9%), pancreas (7.5%), rectum (6.7%) and esophagus (5.8%). On the other hand, the half of neuroendocrine tumor originated rectum (50.9%) and followed by pancreas (13.9%), duodenum (9.0%), lung/bronchus (8.9%), and stomach (8.7%). Neuroendocrine carcinoma presented at more advanced stage and higher age than neuroendocrine tumors.　Most cases of neuroendocrine tumors were treated surgically, while half of neuroendocrine carcinomas were treated with non-surgical therapy consisting of chemotherapy with or without radiotherapy.

**Conclusions:**

Our results demonstrated that neuroendocrine neoplasms could originate from various organs and the site distribution was different between neuroendocrine carcinoma and tumor. The national database of hospital-based cancer registries in Japan is a valuable source for evaluating the organ distribution of the rare systemic disease.

## Introduction

Neuroendocrine neoplasms (NENs) are a form of cancer arising from cells of diffuse neuroendocrine system [1]. NENs are a heterogeneous group of epithelial neoplastic proliferations ranging from indolent well differentiated neuroendocrine tumors (NETs) to very aggressive poorly differentiated neuroendocrine carcinomas (NECs) [2, 3]. Neuroendocrine cells, although a heterogeneous cell population, are characterized by amine and neuropeptide hormone production and dense core vesicles. Despite the diversity in tissue origin, all these tumors share common morphological features, including growth pattern and expression of neuroendocrine markers. Thus, NENs comprise a heterogenous family with wide and complex clinical behaviors according to the primary sites and degreed differentiation of tumor cells [1, 4].

In addition, NENs are essentially rare disease, and the regional differences of primary tumor prevalence has been reported [4–14]. Yao et al. [6] and Hauso et al. [7] summarized the survey study of NENs using the data from the US surveillance, Epidemiology, and end Results (SEER) and the Norweigian Registry of Cancer (NRC), respectively and reported that the incidence rate of NENs increased over the decades. Based on these databases, lung NENs in white population and rectal NENs in black population in SEER were more frequently reported [6], while small intestinal NENs was most frequent in NRC [7]. In addition, several surveys including their studies showed that observed 5-year survival rates were different according to the primary sites of NENs [5–10].

On the other hand, there have been few epidemiological studies of NENs in Japan [11–14]. Ito et al. [11] conducted a nationwide survey of gastroenteropancreatic (GEP) NENs in Japan using a stratified random sampling method. Subsequently, Japan Neuroendocrine Tumor Society (JNETS) examined the distribution of GEP-NENs using data from population-based registry [14]. These studies indicated that the frequency of midgut (jejunum, ileum, and vermiform appendix) NENs in the Japanese population was low compared with Western populations [11–14], suggested that there are ethnic differences in organ distribution of NENs [4–14]. However, Japanese epidemiological results of NENs were focused on GEP-NENs and lacked data on systemic sites of NENs [11–14]. Therefore, a real-world case study of Japanese NENs is necessary to determine the actual clinical information regarding the site distribution. These epidemiological analyses contribute to better understanding of the disease entity and are important to see the regional differences in the world [4–14].

This study was performed to examine the organ distribution of the primary sites, the disease extent and initial treatment of NENs in the Japanese population using a hospital-based cancer registry (HBCR) national database.

## Methods

### Data sources

We retrieved the HBCR national database to identify patients newly diagnosed and treated for NENs between 2009 and 2015. HBCR is mandated to all cancer care hospitals designated by the Ministry of Health, Labor and Welfare in Japan [15, 16]. The designated cancer care hospitals are expected to serve as hubs for providing standard care, including surgery, chemotherapy, and radiotherapy, to cancer patients in their respective regions and to register newly, diagnosed and/or treated cancer cases at their hospitals every year. From 2007, the collected data from designated cancer care hospitals were managed in the Center of Cancer Control and Information Services at the National Cancer Center. Subsequently, non-designated cancer care hospitals that played similar roles in cancer care and HBCR had submitted their HBCR data to the National Cancer Center from 2013. These institutions maintain HBCRs and collect basic information on all newly encountered cancer cases, such as tumor location, histology, route of referral to the hospital, and first-course treatment. All target neoplasms newly encountered at the hospitals are registered. To properly manage the registry, the hospitals are required to employ one or more tumor registrars who have completed a basic training course offered by the National Cancer Center in Japan. The numbers of hospitals included in the HBCR database were 370 in 2009, 387 in 2010, 395 in 2011, 397 in 2012, 409 in 2013, 421 in 2014, and 427 in 2015. A total of 4,263,260 newly diagnosed and treated cancer cases were collected.

In this study, we used the linked database submitted to the National Cancer Center from 2009 to 2015 regarding patients with NENs in Japan. NENs consisted of NETs and NECs in the present study.

### Identification of eligible cases

The definition of malignancy corresponds to behavioral code 2 or 3 in the International Classification of Diseases for Oncology, third edition (ICD-O-3). We collected total 78,069 patients with NENs coded as Class of Cases of 2 (diagnosed and treated in the registering hospital) and 3 (diagnosed in another hospital and treated in the registering hospital). Among the cases, we excluded the cases diagnosed as small cell lung carcinoma (SCLC; code number 80413). Histological Code numbers in NECs included 80,413 (small cell carcinoma, except lung), 80,133 (large cell neuroendocrine carcinoma), 82,433 (goblet cell carcinoid), 82,443 (adenoneuroendocrine carcinoma), 82,453 (adeno carcinoid tumor) and 82,463 (neuroendocrine carcinoma NOS). On contrary, 81,503 (pancreas neuroendocrine tumor), 82,403 (carcinoid NOS), 82,413 (enterochromaffin cell carcinoid), 82,493 (atypical carcinoid), 81,513 (insulinoma), 81,523 (enteroglucagonoma), 81,533 (gastrinoma), 81,543 (mixed pancreatic neuroendocrine-non-neuroexocrine tumor), 81,553 (vipoma) and 81,563 (somatostatinoma) were classed as histological types of NETs. Among them, 81,513 (insulinoma), 81,523 (enteroglucagonoma), 81,533 (gastrinoma), 81,543 (mixed pancreatic neuroendocrine-non-neuroexocrine tumor), 81,553 (vipoma) and 81,563 (somatostatinoma) were defined as functional NETs in the present study. UICC stages with TNM (6^th^ edition until 2011 and 7^th^ edition after 2012 year) was used for stage classification.

### Analysis

We collected the registered cases in HBCR histologically confirmed as NENs in the present study and examined the distribution of the primary sites, age at diagnosis, sex, clinical stage of the disease and initial treatments in patients with NENs. These surveys were compared between NECs and NETs in the present study, respectively. Registry of NENs cases has been done since 2009, however, stage classification of NETs on HBCR system were initiated since 2012. Thus, the data analysis of stage and initial treatment were performed from 2012 to 2015 HBCR database in both NETs and NECs in the present study. The study was carried out in accordance with the Declaration of Helsinki and with Good Clinical Practice guidelines. The dataset was used with permission from the Data Utilization Committee of the Hospital-based Cancer Registry National Registry (National Cancer Center). Furthermore, the study was approved by the Institutional Review Board of Shinshu University School of Medicine (No.4618).

## Results

**Fig. 1:**
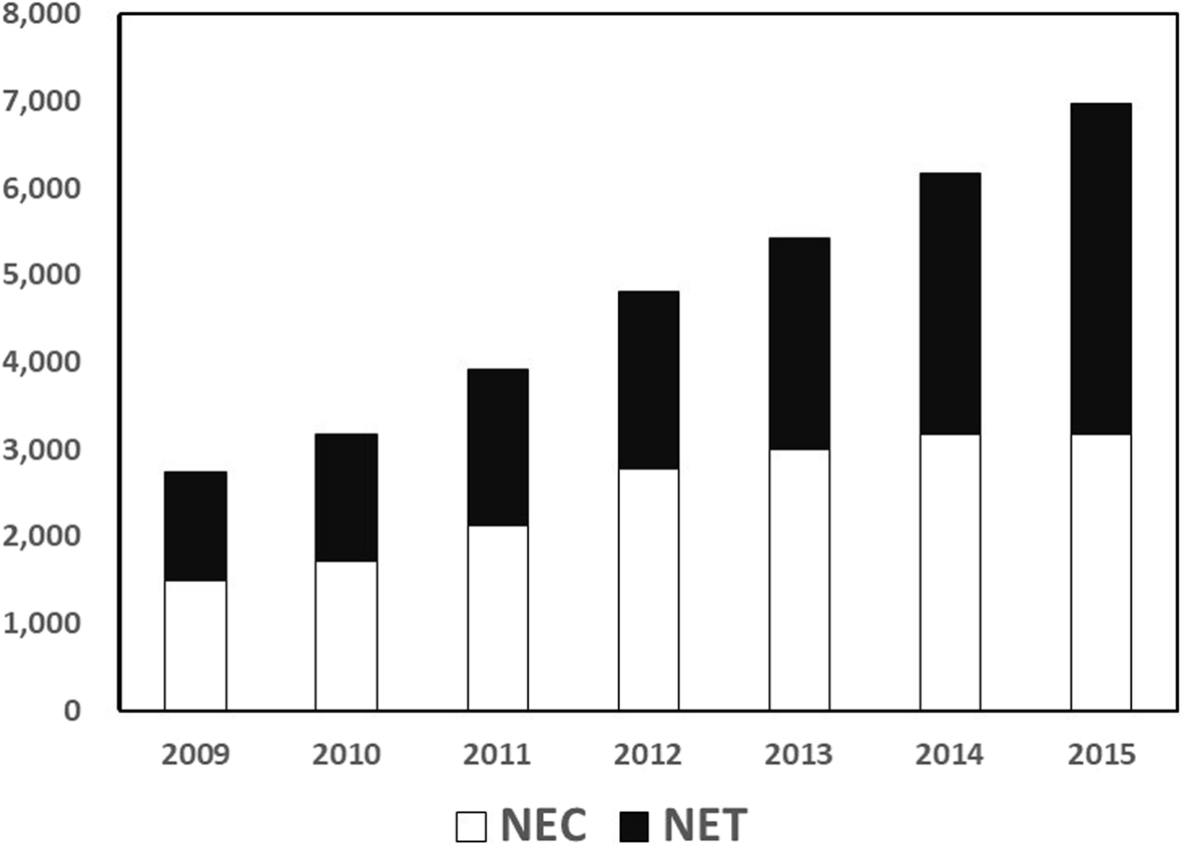
Time courses of changes in number of neuroendocrine carcinoma and tumors. Per year. Black bar: neuroendocrine carcinoma; white bar: neuroendocrine tumor

**Fig. 2:**
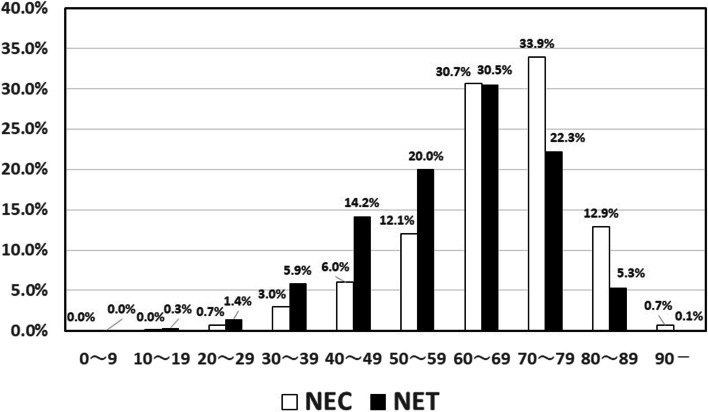
Comparison of age distributions between neuroendocrine carcinoma and tumors. Black bar: neuroendocrine carcinoma; white bar: neuroendocrine tumor

A total case of 33,215 NENs, consisting of 17,485 (52.6%) of NECs and 15,730 (47.4%) of NETs, were diagnosed between 1 January 2009 and 31 December 2015. The number of NECs and NETs cases per year are shown in Fig. 1. A total of 1501 cases of NEC were registered in 2009 and increased gradually to 3178 cases in 2015. A total 1237 of NET was registered in 2009 and increased gradually to 3798 cases in 2015. Thus, both NECs and NETs increased over time gradually. The age distributions of NECs were higher than those in NETs (Fig. 2) with the median ages of 69.0 years old in NECs and 62.0 years old in NETs, respectively. Regarding to the sex distribution, men were dominant in both groups, 67.9% in NECs and 58.1% in NETs, respectively.

Histological subtype in NECs included neuroendocrine carcinoma (48.4%), large cell neuroendocrine carcinoma (30,6%) and small cell carcinoma (17.1%). Main histological types in NETs were carcinoid (86.1%) and atypical carcinoid (8.7%).

### Primary tumor site

The organ distribution of primary sites in NECs and NETs patient are summarized in Tables 1 and 2, respectively. Both NECs and NETs, were distributed widely throughout the body. In NECs (Table 1), the majority occur in lung (31.1%) followed in decreasing frequency by stomach (12.9%), pancreas (7.5%), rectum (6.7%) and esophagus (5.8%). The remaining other organs in NECs included uterus, oral cavity, thymus, mediastinum and ovary et al. On the other hand, the half of NETs patients in Japan was rectum (50.9%) and followed by pancreas (13.9%), duodenum (9.0%), lung/bronchus (8.9%), and stomach (8.7%). Other organs included breast, larynx, esophagus and bladder et al. The locations of the primary tumors varied by sex; compared with female, male patients were more likely in lung, stomach, esophagus and pancreas in NECs, whereas male patients were more likely in the rectum duodenum, thymus, jejunum/ileum, in NETs. In total NENs, the most frequent site was rectum (27.6%), followed by lung (20.7%), stomach (10.9%), pancreases (10.6%), duodenum (5.0%) and colon (3.9%).

**Table 1:** Distributions of neuroendocrine carcinoma by primary sites, Japan, 2009–2015

**Sites**	**ALL**		**Male**		**Female**	
	**Number**	**%**	**Number**	**%**	**Number**	**%**
**Lung**	**5,465**	**31.3%**	**4,626**	**26.5%**	**839**	**4.8%**
**Stomach**	**2,256**	**12.9%**	**1,813**	**10.4%**	**443**	**2.5%**
**Pancreas**	**1,315**	**7.5%**	**776**	**4.4%**	**539**	**3.1%**
**Rectum**	**1,174**	**6.7%**	**738**	**4.2%**	**436**	**2.5%**
**Esophagus**	**1,019**	**5.8%**	**783**	**4.5%**	**236**	**1.3%**
**Colon**	**983**	**5.6%**	**527**	**3.0%**	**456**	**2.6%**
**Cervix**	**752**	**4.3%**		**0.0%**	**752**	**4.3%**
**Bladder**	**577**	**3.3%**	**430**	**2.5%**	**147**	**0.8%**
**Bile cystic duct**	**480**	**2.7%**	**243**	**1.4%**	**237**	**1.4%**
**Breast**	**429**	**2.5%**	**7**	**0.0%**	**422**	**2.4%**
**Prostate**	**289**	**1.7%**	**289**	**1.7%**		**0.0%**
**Duodenum**	**245**	**1.4%**	**157**	**0.9%**	**88**	**0.5%**
**Others**	**2,501**	**14.3%**	**1,491**	**8.5%**	**1,010**	**5.8%**
**Total**	**17,485**	**100.0%**	**11,880**	**67.9%**	**5,605**	**32.1%**

**Table 2:** Distributions of neuroendocrine tumors by primary sites, Japan, 2009–2015

**Sites**	**ALL**		**Male**		**Female**	
	**Number**	**%**	**Number**	**%**	**Number**	**%**
**Rectum**	**8,003**	**50.9%**	**4,908**	**31.2%**	**3,095**	**19.7%**
**Pancreas**	**2,190**	**13.9%**	**1,064**	**6.8%**	**1,126**	**7.2%**
**Duodenum**	**1,418**	**9.0%**	**902**	**5.7%**	**516**	**3.3%**
**Lung**	**1,406**	**8.9%**	**721**	**4.6%**	**685**	**4.4%**
**Stomach**	**1,373**	**8.7%**	**801**	**5.1%**	**572**	**3.6%**
**Colon**	**320**	**2.0%**	**173**	**1.1%**	**147**	**0.9%**
**Small intestines**	**258**	**1.6%**	**178**	**1.1%**	**80**	**0.5%**
**Thymus**	**142**	**0.9%**	**105**	**0.7%**	**37**	**0.2%**
**Bile cystic duct**	**129**	**0.8%**	**70**	**0.4%**	**59**	**0.4%**
**Ovarium**	**94**	**0.6%**		**0.0%**	**94**	**0.6%**
**Mediastinum**	**65**	**0.4%**	**44**	**0.3%**	**21**	**0.1%**
**Liver**	**64**	**0.4%**	**30**	**0.2%**	**34**	**0.2%**
**Others**	**268**	**1.7%**	**141**	**0.9%**	**127**	**0.8%**
**Total**	**15,730**	**100.0%**	**9,137**	**58.1%**	**6,593**	**41.9%**

Total 255 cases (136 in male, 119 in female) of functional NETs were newly diagnosed (2009–2015) and registered HBCR national database in the present study, comprised 1.6% in NETs. The primary site was pancreas (207 cases, 82%), followed by duodenum (32 cases), bile duct/liver (6 cases) and stomach (3 cases) et al.

### Stages distribution and initial therapies

**Fig. 3:**
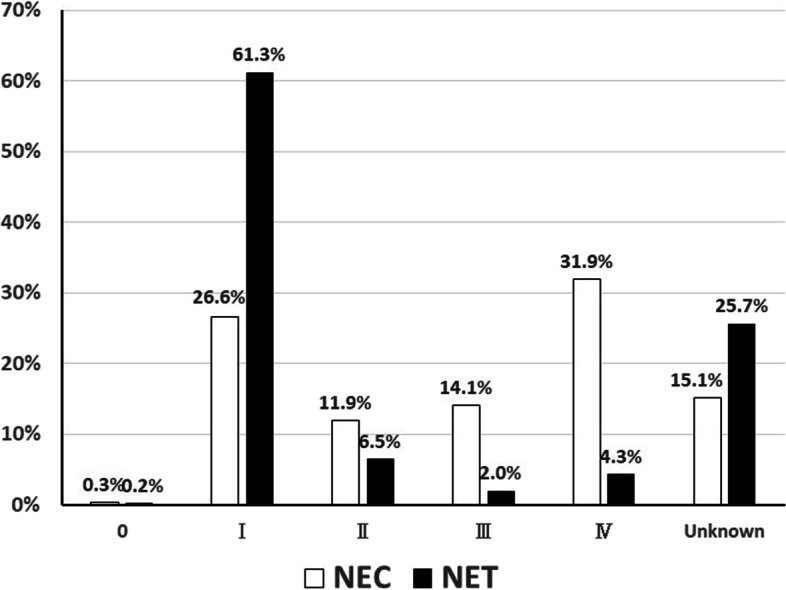
Stage distribution of the disease in neuroendocrine carcinoma and tumors from 2012 to 2015 in the present study. Black bar: neuroendocrine carcinoma; white bar: neuroendocrine tumor

Staging distributions data in NECs and NETs from 2012 to 2015 was shown in Fig. 3. The most frequent stage in NECs was stage IV (31.9%) and followed by stage I (26.6%), suggested that NECs were diagnosed at advanced stage. On contrary, almost half of NETs (61.3%) were shown to be stage I and the early stage (stage I) was apparently higher than those in other advanced stages. However, 25.7% in NETs and 15.1% in NECs cases were registered as “not evaluated” and/or “unknown data”. Therefore, the HBCR database might be insufficient data on the extent of disease for this analysis.

**Fig. 4:**
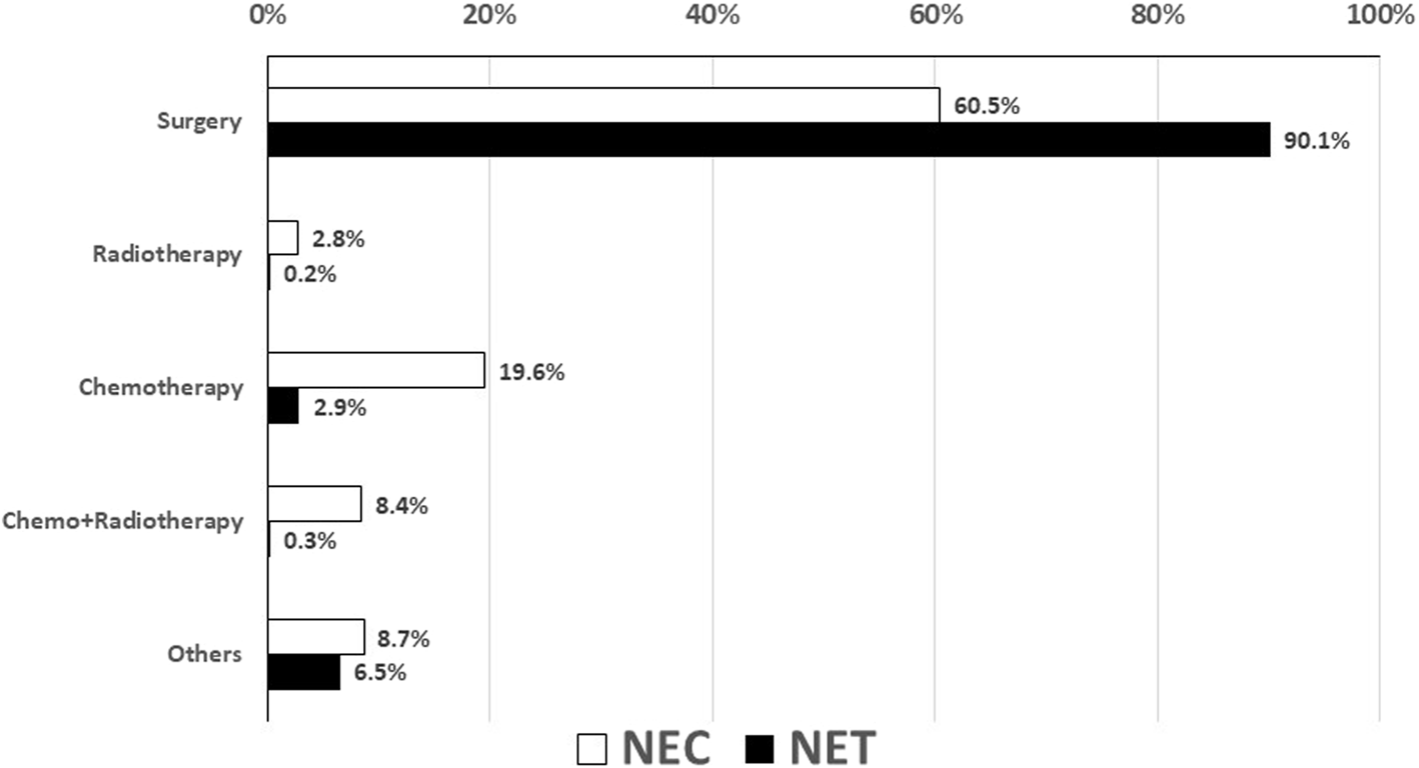
Comparison of initial therapies between neuroendocrine carcinomas and tumors from 2012 to 2015. Black bar: neuroendocrine carcinoma; white bar: neuroendocrine tumor

The initial therapies for NECs and NETs are summarized in Fig. 4. Multimodality therapy, including surgical approaches, was conducted in 60.5% of NECs and 90.1% of NETs patients, respectively. Multimodality therapy including chemotherapy, radiotherapy or the combination without surgery was dominant in NECs (chemotherapy (19.6%), chemoradiotherapy (8.4%) radiotherapy (2.8%), compared with those in NETs.

## Discussion

The present study was the first trial to see the real-world practice of NENs in Japan. The HBCR national database in Japan was estimated to cover 67% of new cancer cases in 2010 [15]. Later, as greater numbers of designated and non-designated cancer care hospitals submitted the HBCR to the National Cancer Center, HBCR database came to cover over 70% of all cancer cases [16, 17]. Although several epidemiological data especially focusing on gastrointestinal and pancreatic NENs in Japan were reported [10–14], we collected the systemic NENs on HBCR in Japan. Indeed, the collected numbers of samples, especially of NETs, were the largest compared with those in previous studies [10–14] in Japan. In addition, to our knowledge, TNM stage distribution and initial treatment in patients with NENs was the first report. Thus, we believed that the epidemiological data of NENs reported here could mirror clinical practice in patients with NENs in Japan.

Median age in NECs was higher than that in NETs in the present study, which were consistent with previous studies [6, 18]. Median age in NECs (69.0 years old) and NETs (62.0 years old) in our data were almost similar with that in SEER data [6, 18] and that in another Japanese gastro-entero-pancreatic NETs [14]. However, median age of NETs in Korea and German was 54.7 and 57 years old, respectively, suggesting that mean age in Japanese NETs was higher than other counties. It has been shown that age distribution varied according to the primary organs in NETs [6, 9, 14]. We need to clarity the age distribution according to the primary sites in NENs in the next study.

Rectum was the most common site and showed a 50.9% of NETs followed by the lung and stomach. A similar finding was detected in several epidemiological studies in Asian counties [10–19]. For examples, epidemiological data of NENs in Taiwan revealed the most common primary sites were rectum (25.4%), lung (20%) and stomach (7.4%) [10]. Regarding to the analysis focusing gastrointestinal and pancreatic NENs, rectum was the most common sites in Japan [11–14], China [19], Korea [20]. In addition, SEER data in Asian/Pacific Islander also showed that the top three primary sites were rectum, lung and pancreas [6]. On contrary, the pancreas and jejunum/ileum were the most frequent positions in German-NET-Registry [9] and the small intestine was the most frequent sites of origin, followed by the colon and rectum in NRC [7]. Thus, organ distribution varied on the racial differences in countries.

Based on the SEER database, lung NENs was most frequent primary site even though SCLC was excluded. In addition, NENs increased the fastest in lung [6] and half of the patients were presented advanced stages [6]. When NETs in thymus and mediastinum were included in lung NETs, “lung” NETs were the third originated site. There was little epidemiological information about “lung” NETs to date, and approved agent was also limited, especially in Japan. Based on our survey, we emphasize that lung NEC and/or NET should be recognized as non-negligible disease.

There were several limitations in the present study. The histological distinction between lung NECs and SCLC has been actually difficult, especially when using only cytology samples. SCLC was excluded in the present study, however, lung NECs might include SCLC in registered system and we might overestimate the number of lung NECs in the present study. Thus, there is a possibility that the organ distribution of lung NECs might be overestimated in the present study, which was the major limitation in the present study. Second, the WHO revised the nomenclature and classification of all NENs as malignancy in 2010 [21] and the stage classification of NETs were registered on HBCR system since 2012. Thus, the stage registry in the present study was insufficient data, which were unable to evaluate the relationship between precise stage and first-line treatment in patients NENs.

Based on cancer registry data, it is evident that NENs incidence in certain countries has increased in the past decades [4–8]. The registered number of NENs in Japan was increased over time and the number of newly participating hospitals submitting HBCR data to the National Cancer Center also increased during the study. However, HBCR national database did not cover the absolute numbers of all NENs patients in Japan. In addition, specialized hospitals for caring patients with NENs were not always included in designated cancer care hospitals. Thus, our data was unable to clarity the incidence rate and the trend in NENs in Japan. Because of its rarity of the disease, there are unmet needs in patients with NENs [22]. Further clinical studies in NENs including the epidemiology are needed.

In summary, based on a hospital-based registry database, our study demonstrated real-world organ distribution in Japanese neuroendocrine neoplasms. The neuroendocrine neoplasms could originate from various organs. The half of all patients with neuroendocrine tumors was rectum in Japan, however, pulmonary (lung and mediastinal) neuroendocrine tumors were also non-negligible disease. Neuroendocrine carcinomas presented at advanced stage and higher age than neuroendocrine tumors. The national database of hospital-based cancer registries in Japan is a valuable source for evaluating the organ distribution of the rare systemic disease.

## Data Availability

The datasets generated during this study are available from the corresponding author on reasonable request on reasonable request.
